# Improved polygenic risk prediction in migraine-first patients

**DOI:** 10.1186/s10194-024-01870-8

**Published:** 2024-09-27

**Authors:** Dora Torok, Peter Petschner, Daniel Baksa, Gabriella Juhasz

**Affiliations:** 1 Department of Pharmacodynamics, Faculty of Pharmaceutical Sciences, Semmelweis University, Nagyvarad ter 4., Budapest, 1096 Hungary; 2 NAP3.0-SE Neuropsychopharmacology Research Group, Hungarian Brain Research Program, Semmelweis University, Budapest, Hungary; 3 Bioinformatics Center, Institute of Chemical Research, Kyoto University, Gokasho, Uji, Kyoto, Japan; 4 Department of Personality and Clinical Psychology, Institute of Psychology, Faculty of Humanities and Social Sciences, Pazmany Peter Catholic University, Budapest, Hungary

**Keywords:** Migraine, Genetics, Missing heritability, Disease onset

## Abstract

**Background:**

Recent meta-analyses estimated 14.6% and 11.2% SNP-based heritability of migraine, compared to twin-heritability estimates of 30–60%. This study aimed to investigate heritability estimates in “migraine-first” individuals, patients for whom G43 (migraine with or without aura) was their first medical diagnosis in their lifetime.

**Findings:**

Using data from the UK Biobank (*N* = 199,929), genome-wide association studies (GWAS) were conducted on 6,139 migraine-first patients and 193,790 healthy controls. SNP-based heritability was estimated using SumHer, yielding 19.37% (± 0.019) for all SNPs and 21.31% (± 0.019) for HapMap3 variants, substantially surpassing previous estimates. Key risk loci included *PRDM16*, *FHL5*, *ASTN2*, *STAT6*/*LRP1*, and *SLC24A3*, and pathway analyses highlighted retinol metabolism and steroid hormone biosynthesis as important pathways in these patients.

**Conclusions:**

The findings underscore that excluding comorbidities at onset time can enhance heritability estimates and genetic signal detection, significantly reducing the extent of “missing heritability” in migraine.

**Supplementary Information:**

The online version contains supplementary material available at 10.1186/s10194-024-01870-8.

## Introduction

Previous large meta-analyses [1, 2] (henceforth, Gormley et al., Hautakangas et al.) estimated 14.6% and 11.2% single nucleotide polymorphism (SNP)-based heritability in migraine (with or without aura), respectively. In contrast, twin-heritability estimates of the disorder range from 30 to 60% [3]. Differences between SNP-based and twin-heritability estimates are considered “missing heritability”, and are often attributed to various factors, like rare variants, environmental factors, or their epigenetic interactions. Some of these factors could be compensated during analyses.

A prominent example can be known comorbidities upon onset. We have previously identified the comorbidity network of migraine. Among others, a factor in this comorbidity map of the disorder was major depression [4]. A later study from our lab using machine learning methods provided substantial evidence that already diagnosed, comorbid major depression can influence relevance and effect size of genomic hits for migraine [5]. Another study reported altered heritability in the presence of comorbid depression [6]. Taken together, findings suggest, given the above are true for other comorbidities too, that migraine-first diagnosis may have a better characterizable genomic background. Thus, to investigate the biological background of the migraine-first diagnosis is important to improve our understanding of migraine pathophysiology and to enhance migraine-specific drug target prediction, particularly as clinical drug trials mainly focus on migraine patients without other comorbidities.

Accurate migraine definition may also improve GWAS results and heritability estimates. Meta-analyses (Gormley et al., and Hautakangas et al.), to include as many individuals as possible and increase statistical power, often use data from self-reported questionnaires to identify participants with migraine. A clinical diagnosis or more homogeneous patient subgroups can provide more homogeneous samples and reduce noise for genomic methods.

In light of the above, our aim was to define a migraine-first population, i.e. migraine patients diagnosed with the disorder (G43, migraine with or without aura) without apparent comorbidities at the time of the diagnosis (henceforth: migraine-first patients) and run genome-wide association study and heritability estimation comparing them to age-matched controls without diagnosed diseases. Our goal was to test if such an approach is capable to discover relevant top hits or increase background polygenic signal in migraine.

## Methods

Data used in our analysis were part of the UK Biobank (application number 71718). Genomic quality control steps and filtering were performed as described in the supplementary information of Eszlari et al. [7], including filtering for minor allele frequency, Hardy-Weinberg equilibrium, missingness, info and certainty filters, linkage disequilibrium pruning, kinship check, sex check, and heterozygosity outlier detection, leaving 334,125 participants with valid genomic data.

Migraine-first patients (*N* = 6,139) were identified by the date of their migraine diagnosis (UKB data-field 131052 - Date G43 first reported [migraine]), with the criterion that migraine was their initial diagnosis. Healthy controls (*N* = 193,790) were defined as individuals who had no recorded diseases at the same age (22 ± 12 years, mean ± standard deviation) when the migraine-first patients received their diagnosis and had no migraine diagnosis in their lifetime. Descriptive statistics were calculated with R (v 4.1.2) and can be found in Supplementary Table S1.

Genome-wide association analyses were performed with PLINK2 [8] on migraine-first patients and healthy controls. After quality control steps and exclusion of SNPs on chromosome X, 6,077,313 SNPs remained. The following covariates were included in the analyses: sex, age (UKB data-field 31 and 21003), first 10 principal components of the genome and genotype array (UKB Axiom Array and Affymetrix UK BiLEVE Axiom Array).

Evaluation of the GWAS result (risk loci identification, gene-set enrichment analysis) was performed with FUMA [9] and MAGMA [10]. Index variants were defined as LD-independent variants (r^2^ < 0.1) and loci containing all variants in high linkage disequilibrium (r^2^ > 0.6) within 250 kb distance to the index variants. (The definitions were the same as in Gormley et al. and Hautakangas et al.). We used two thresholds for lead SNP identification: a genome-wide significance (GWS) value of *p* < 10^− 8^_,_ and a suggestive significance of 10^− 8^ < *p* < 10^− 5^.

SNP-based heritability was estimated with SumHer (part of the software package LDAK, version 5.2.) [11], following the authors’ recommendation, using a one-parameter heritability model - The Human Default Model (specified by adding these parameters: --ignore weights YES and --power − 0.25) for (1) all SNPs, (2) all SNPs with genome-wide (GWS) and suggestive significant SNPs excluded, and (3) SNPs restricted for the HapMap3 SNP-set. MHC region (chr 6, 25–35 Mb) was excluded from FUMA and MAGMA analyses and all heritability estimations. The estimated heritability was converted to liability scale using 0.03 “ascertainment” (reflecting that migraine proportion was 0.03 in our sample) and “prevalence” was set to 0.16 (as in Hautakangas et al.). During analyses no genomic inflation correction was used to be able to compare results with Gormley et al. and Hautakangas et al., and follow the guidelines of the authors of SumHer. In addition, such a correction is recommended only for low quality data with population structure and relatedness, which we controlled due to the standard quality control pipeline employed.

For validation purposes, we also tested (1) our control group definition by performing the same analysis steps with controls without restrictions (henceforth: all participants without migraine diagnosis in their lifetime, (*N*_all_controls_ = 327,986, Supplementary Table S2), (2) the influence of the small proportion of migraine patients in our population (ascertainment) by running and averaging three analyses after excluding a third of randomly selected healthy controls to increase the “ascertainment” parameter (Supplementary Table S3), (3) our phenotype definition by conducting the same analysis steps for all migraine diagnosed patients (G43) without filtering for first onset (*N*_G43_diagnosis_ = 17,679, *N*_all_controls_ = 316,446, Supplementary Table S4), (4) the effect of the heritability model [11–13] namely, genome-wide complex trait analysis (GCTA) using the following parameters: --ignore weights YES and --power 1; Human Default Model (HD) with --ignore weights YES and --power − 0.25; LDAK model with --weights < weights for each variant > and --power − 0.25; and BLD-LDAK model with --annotation-number 65, --annotation-prefix < baseline annotation categories>, --ignore-weights YES and --power − 0.25. All model assumptions are described in Supplementary Table S6.

In addition, to assess whether including variants with a lower MAF (MAF ≥ 0.001) could enhance heritability estimation, we expanded our analysis to include these rare variants (MAF extended analysis).

## Results

**Fig. 1:**
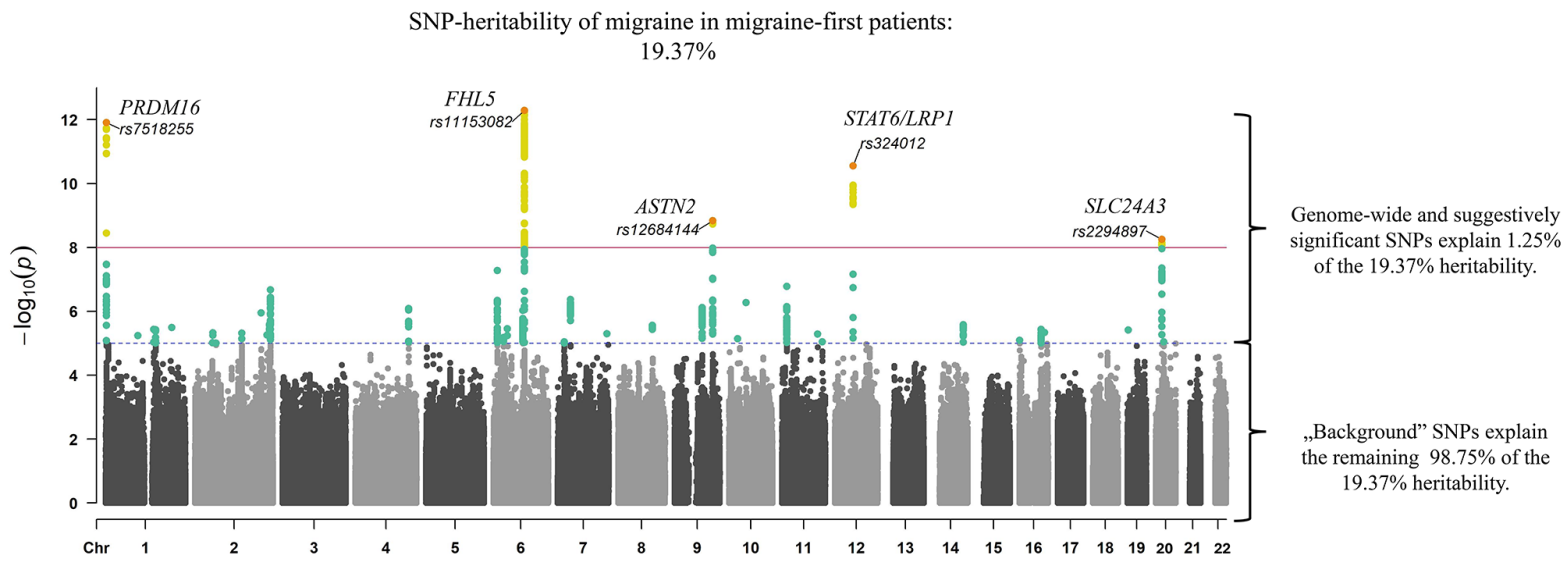
Summary of the results: genome-wide significant loci and SNP-heritability of migraine-first patients and healthy controls

Figure 1 shows the five genome-wide significant risk loci identified both in our GWAS, in the study of Gorlmey et al., and Hautakangas et al. The red line denotes genome-wide significance, and the blue dashed line denotes the suggestive significance threshold. Yellow dots are genome-wide significant, and green dots are suggestively significant SNPs. The colored SNPs denote the combination of genome-wide- and suggestively significant SNPs (explaining 1.25% of the total heritability). In contrast, black and grey dots denote the “background” SNPs, explaining a large majority of the total heritability (98.75%). Migraine-first patients: initial filtering was based on G43 migraine (with or without aura) diagnosis and narrowed down to migraine-first patients for whom G43 (migraine with or without aura) was their first medical diagnosis in their lifetime. Healthy controls: individuals who had no recorded diseases at the same age when the migraine-first patients received their diagnosis and had no migraine diagnosis in their lifetime.

SumHer estimated SNP-based heritability was 19.37% (± 0.019, SD) for migraine in migraine-first patients using all SNPs (Fig. 1). Excluding genome-wide and suggestively significant hits still resulted in a SNP-based heritability of 18.13% (± 0.019, SD), indicating that the majority of the signal stems from the background polygenic signal. Restricting our SNP set to HapMap3 variants, in accordance with Gormley et al. and Hautakangas et al., gave a SNP-based heritability of 21.31% (± 0.019, SD). This is a 45.96% and 90.27% increase compared to the study of Gormley et al. and Hautakangas et al., respectively. All heritability results can be found in Supplementary Table S5.

**Table 1:** Genome-wide and suggestively significant SNPs and risk loci

**GWS SNPs**				
	Current study	Gormley et al.	Hautakangas et al.	Overlap in all three
GWS SNPs	10	44(1)	170(n/a)	-
GWS risk loci	5	38(5)	123(5)	5
**GWS + suggestive SNPs**				
	Current study	Gormley et al.	Hautakangas et al.	Overlap in all three
GWS + suggestive SNPs	64	44(5)	170(n/a)	-
GWS + suggestive risk loci	36	38(11)	123(11)	11

Table 1 shows the overlapping SNPs and loci based on the results of Gormely et al., and Hautakangas et al., and our current study. Numbers in brackets denote the overlap with our current study. In case of Hautakangas et al., only risk loci data was available. GWS: genome-wide significant (*p* < 10^− 8^), GWS + suggestive: significance threshold is *p* < 10^− 5^, n/a -not available.

Full list of GWS and suggestively significant SNPs can be found in Supplementary Tables S9-S10. Among risk loci calculated using GWS SNPs (Supplementary Table S11), 100% of our present results were found in the previous meta-analyses. With suggestive significance threshold (to avoid a reduction in potential risk loci caused by reduced power in the current study, Supplementary Table S12), 30.56% of risk loci replicated in all studies (see Table 1). Prominent genes associated with GWS and suggestive significant risk loci were identified as: *PRDM16*, *FHL5*, *ASTN2*, *STAT6*/*LRP1*, *SLC24A3*, and the former five genes plus *ADAMTSL4*, *MEF2D*, *TRPM8*, *PHACTR1*, *SUGCT/C7orf10*, *ITPK1*, respectively.

We could explicitly replicate significant tissue-specific enrichment of our risk loci in tibial artery tissues (Supplementary Table S13), similarly to both Gormley et al. and Hautakangas et al. Other tissue-specific enrichment remained non-significant.

Using risk loci based on GWS SNPs, pathway-level analysis yielded no significant gene sets, while employing suggestive significant SNPs gave seven KEGG pathways (MsigDB C2 sets), including e.g., retinol metabolism and steroid hormone biosynthesis, among others (Supplementary Figure S2). Enrichment of MsigDB C3 sets resulted in STAT1 and PEA3 significant transcription factor-related sets (Supplementary Figure S3).

Validation efforts showed (Supplementary Table S5) that (1) although we observed a higher statistical power with more significant GWS hits increasing the number of controls, the heritability estimate was lower (h^2^ = 17.39%, a 10.22% reduction on liability scale), (2) employing more balanced samples with higher proportion of migraine patients compared to controls decreased SNP-based heritability (h^2^_mean_ = 18.45%, a 13.42% reduction on liability scale), probably due to the reduced statistical power, but still remained substantially higher than in previous studies, (3) heritability of G43 migraine diagnosis without filtering for first onset was low (h^2^ = 12.92%, a 39.32% reduction on liability scale) (4) the impact of GCTA heritability model was small and yielded comparable results (h^2^ = 23.89%) to HD. Results for all other heritability estimation analysis can be found in Supplementary Tables S5, S7 and S8. Results regarding MAF extended analyses showed that extending our analyses to include rarer variants can enhance the explained heritability, albeit, average contribution of the rarer variants was in general smaller compared to more common ones (Supplementary Table S7, Supplementary Fig. 1).

All in all, validation efforts indicated that control group definition, ascertainment parameter and heritability model could not explain the elevated SNP-based heritability in migraine-first patients, while the diagnosis alone without considering first onset on the UKB population yielded comparable heritability to Gormley et al. Although there was a modest enhancement in heritability estimation, the overall contribution of variants with lower MAF appears to be low.

## Discussion

In our study, substantially elevated SNP-based heritability and replicable top hits in risk loci of *PRDM16*, *FHL5*, *ASTN2*, STAT6/*LRP1*, *SLC24A3* genes were found among migraine-first patients (Fig. 1).

Previous meta-analyses identified 38 and 123 risk loci with significance values reaching 5.6 × 10^− 49^ and 10^− 90^ in 59,674 cases and 316,078 controls, and 102,084 cases and 771,257 controls in the studies of Gormley et al. and Hautakangas et al., respectively. The two meta-analyses also indicated a 14.6% and 11.2% SNP-based heritability of migraine.

Using migraine-first patients, we found a 45.96% and 90.27% increase in SNP-based heritability with a much smaller sample size. From our estimated 19.37% heritability, top hits (at a GWS and suggestive significance level) explained only 1.25% (Fig. 1), which is well in line with the polygenic structure of migraine and suggests that an elevated background polygenic signal is primarily responsible for the observed increase in SNP-based heritability. In addition, 100% of our risk loci overlapped with results of previous meta-analyses in case of GWS- (5/5, Table 1) and 31% in case of suggestive significant hits (11/36, Table 1), albeit, the most significant risk locus obtained a significance of only 10^− 13^. The identified risk loci are related to (1) thermoregulation, adult neurogenesis, and cerebrospinal fluid maintenance (*PRDM16*); (2) vascular remodeling and gene programs (*FHL5*); (3) neuronal migration and schizophrenia (*ASTN2*); (4) vitamin A transport into immune cells, Alzheimer’s disease, lipid homeostasis, various vascular disorders, spontaneous coronary artery dissection [14, 15], in cervical artery dissection [16], in abdominal aortic aneurysm [17, 18], in descending aorta strain and, in descending aortic diameter [19] (*LRP1*); (5) sodium-calcium change and intracellular calcium homeostasis (*SLC24A3)* (www.genecards.org, https://www.ebi.ac.uk/gwas/) [5, 20]. Among the pathways, STAT1-, PEA3- and retinol metabolism-related sets implicate immunologic and vitamin A-bound processes behind migraine. Tissue-specific enrichment in tibial artery confirmed previous assumptions about vascular components of the disease, which is in line with LRP1-associated processes. In sum, significant findings were consistent with previous studies.

Although large meta-analyses with less well-defined phenotypes possess large statistical power and can identify top hits in migraine with high significance, they seem to substantially underestimate the background polygenic signal based on the current results. This phenomenon may distort the picture of the real underlying genetic architecture of migraine, overemphasizing top hits and underemphasizing the underlying background polygenic signal. In accordance, results also show that these top hits do contribute, but a larger part of the genomic signal stems from non-significant hits. Given these considerations, polygenic risk scores that estimate such polygenicity and could be useful in disease and prognosis prediction may be calculated more accurately from better-defined, more homogenous patient subgroups, like our migraine-first group.

All in all, our study provided evidence that migraine patients who had their migraine diagnosis first have higher heritability compared to a general migraine population. Additionally, this migraine-first subgroup also showed association with the same genetic risk factors that were established based on large heterogeneous cohorts, but the top hits could only explain a small proportion of this heritability. Three clinical implications emerge: (1) optimized and more precise estimation of the polygenic risk of migraine based on migraine-first patients can pave the way for improved prediction of migraine risk, (2) key research attempts to find novel migraine medications could be improved by further investigating biological mechanisms related to the polygenic background of patients with a migraine-first diagnosis, (3) and finally, due to the fact that migraine heritability mainly explained by polygenicity, novel migraine treatment strategies probably benefit from a multi-target approach.

## Electronic supplementary material

Below is the link to the electronic supplementary material.

Supplementary Material 1

## Data Availability

No datasets were generated or analysed during the current study.
