# Supplementary material for: Improved polygenic risk prediction in migraine-first patients
Source: J Headache Pain. 2024 Sep 27;25(1):161. doi: 10.1186/s10194-024-01870-8 (PMC11438044; doi:10.1186/s10194-024-01870-8)
Supplement: Supplementary file 1 — Supplementary Material 1 [file 10194_2024_1870_MOESM1_ESM.pdf]

Improved Polygenic Risk Prediction in Migraine-First Patients

Dora Torok[1,2,†], Peter Petschner[1,2,3,†], Daniel Baksa[1,2,4], Gabriella Juhasz[1,2*]

[1] Department of Pharmacodynamics, Faculty of Pharmaceutical Sciences, Semmelweis University, Budapest, Hungary

[2] NAP3.0-SE Neuropsychopharmacology Research Group, Hungarian Brain Research Program, Semmelweis University, Budapest, Hungary

[3] Bioinformatics Center, Institute of Chemical Research, Kyoto University, Gokasho, Uji, Kyoto, Japan

[4] Department of Personality and Clinical Psychology, Institute of Psychology, Faculty of Humanities and Social Sciences, Pazmany Peter Catholic University, Budapest, Hungary

[*] corresponding author: Gabriella Juhasz, Department of Pharmacodynamics, Faculty of Pharmaceutical Sciences, Semmelweis University, Nagyvarad ter 4., Budapest, Hungary, 1096.
Email: juhasz.gabriella@semmelweis.hu

[†] - indicates shared first authorship

Supplementary Material

Table of contents

1. Population description 3
1.1. Migraine-first patients and healthy controls 3
1.2. Migraine-first patients and all controls 4
1.3. Migraine-first patients and a third of randomly selected healthy controls – datasets 1-3 5
1.4. G43 migraine-diagnosed patients without considering first onset and all controls 6
2. Heritability 7
2.1. Results of validation heritability estimations 7
2.2. Heritability models 8
2.3. Results of different heritability models 9
3. Results of genome-wide association studies of migraine-first patients and healthy controls 11
3.1. Genome-wide significant SNPs 11
3.2. Suggestively significant SNPs 12
3.3. Genome-wide significant risk loci 13
3.4. Genome-wide and suggestively significant risk loci 14
3.5. GTEx v8 significant tissue type 15
3.6. Gene-set enrichment analysis 15
3.6.1. MsigDB C2 – KEGG 15
3.6.2. MsigDB C3 – Transcription Factor Targets 16

1. Population description

1.1. Migraine-first patients and healthy controls

Supplementary Table S1

Migraine-first patients and healthy controls		
Age	Mean	58.1743
	SD	±7.4741
Sex	Females	101766 (51%)
	Males	98163 (49%)
Migraine-first cases – healthy controls	Cases	6139 (3%)
	Controls	193790 (97%)
Migraine-first patients - age	Mean	56.0664
	SD	±7.7387
Migraine-first patients - sex	Females	4688 (76%)
	Males	1451 (24%)
Healthy controls - sex	Females	97078 (50%)
	Males	96712 (50%)
Healthy controls - age	Mean	58.2410
	SD	±7.4559
Age when migraine was diagnosed (in migraine-first patients)	Mean	22.2105
	SD	±12.2849

Supplementary Table S1 Descriptive statistics of migraine-first patients and healthy controls. SD – standard deviation.

Migraine-first patients: initial filtering was based on G43 migraine (with or without aura) diagnosis and narrowed down to migraine-first patients for whom G43 (migraine with or without aura) was their first medical diagnosis in their lifetime.

Healthy controls: individuals who had no recorded diseases at the same age when the migraine-first patients received their diagnosis and had no migraine diagnosis in their lifetime.

1.2. Migraine-first patients and all controls

Supplementary Table S2

Migraine-first patients and all controls		
Age	Mean	56.8738
	SD	±7.9919
Sex	Females	179427 (54%)
	Males	154698 (46%)
Migraine-first patients – all controls	Cases	6139 (2%)
	Controls	327986 (98%)
Migraine-first patients - sex	Females	4688 (76%)
	Males	1451 (24%)
All controls - sex	Females	174739 (53%)
	Males	153247 (47%)
Migraine-first patients - age	Mean	56.0665
	SD	±7.7387
All controls - age	Mean	56.8889
	SD	±7.9958

Supplementary Table S2 Descriptive statistics of the 1st validation analysis, migraine-first patients, and all controls. SD – standard deviation.

Migraine-first patients: initial filtering was based on G43 migraine (with or without aura) diagnosis and narrowed down to migraine-first patients for whom G43 (migraine with or without aura) was their first medical diagnosis in their lifetime.

All controls: all participants without migraine diagnosis in their lifetime.

1.3. Migraine-first patients and a third of randomly selected healthy controls – datasets 1-3

Supplementary Table S3

Dataset 1		
Age	Mean	58.1508
	SD	±7.4857
Sex	Females	69375 (51%)
	Males	65958 (49%)
Migraine-first patients – randomly selected healthy controls	Cases	6139 (5%)
	Controls	129194 (95%)
Dataset 2		
Age	Mean	58.1678
	SD	±7.4784
Sex	Females	69431 (51%)
	Males	65902 (49%)
Migraine-first patients – randomly selected healthy controls	Cases	6139 (5%)
	Controls	129194 (95%)
Dataset 3		
Age	Mean	58.1271
	SD	±7.4866
Sex	Females	69368 (51%)
	Males	65965 (49%)
Migraine-first patients – randomly selected healthy controls	Cases	6139 (5%)
	Controls	129194 (95%)

Supplementary Table S3 Descriptive statistics of the 2nd validation analysis, three datasets with randomly selected healthy controls. SD – standard deviation.

Migraine-first patients: initial filtering was based on G43 migraine (with or without aura) diagnosis and narrowed down to migraine-first patients for whom G43 (migraine with or without aura) was their first medical diagnosis in their lifetime.

Randomly selected healthy controls: three datasets of a third (129,194) of the healthy controls (individuals who had no recorded diseases at the same age when the migraine-first patients received their diagnosis and had no migraine diagnosis in their lifetime) to increase the "ascertainment" parameter.

1.4. G43 migraine-diagnosed patients without considering first onset and all controls

Supplementary Table S4

Patients diagnosed with G43 and all controls		
Age	Mean	56.8738
	SD	±7.9919
Sex	Females	179427 (54%)
	Males	154698 (46%)
G43 migraine - all controls	Cases	17679 (5%)
	Controls	316446 (95%)
G43 migraine patients - age	Mean	55.5428
	SD	±7.8920
G43 migraine patients - sex	Females	13228 (75%)
	Males	4451 (25%)
All controls - sex	Females	166199 (53%)
	Males	150247 (47%)
All controls - age	Mean	56.9482
	SD	±7.9910
Age when migraine was diagnosed (for G43 migraine patients)	Mean	37.1125
	SD	±18.9314

Supplementary Table S4 Descriptive statistics of the 4th validation analysis, total UK Biobank population for all G43 migraine diagnosed patients without filter for first onset and all controls. SD – standard deviation.

G43 migraine patients: all migraine-diagnosed patients (G43) without filtering for first onset.

All controls: all participants without migraine diagnosis in their lifetime.

2. Heritability

2.1. Results of validation heritability estimations

Supplementary Table S5

**Migraine-first cases and healthy controls**	**Heritability on liability scale**	**SD ±**
1)All SNPs	19.37%	0.019
2)All SNPs with GWS and suggestive significant SNPs excluded	18.13%	0.019
3)SNPs restricted for the HapMap3 SNP-set	21.31%	0.019
**Validation model 1: migraine-first cases and all controls**	**Heritability on liability scale**	**SD ±**
All SNPs	17.39%	0.016
**Validation model 2: migraine-first patients and randomly selected healthy controls - dataset 1**	**Heritability on liability scale**	**SD ±**
SNPs restricted for the HapMap3 SNP-set	18.54%	0.017
**Validation model 2: migraine-first patients and randomly selected healthy controls - dataset 2**	**Heritability on liability scale**	**SD ±**
SNPs restricted for the HapMap3 SNP-set	18.18%	0.017
**Validation model 2: migraine-first patients and randomly selected healthy controls - dataset 3**	**Heritability on liability scale**	**SD ±**
SNPs restricted for the HapMap3 SNP-set	18.63%	0.017
Mean of the heritability of the 3 validations	18.45%	0.017

**Validation model 3: effect of the heritability model (GCTA versus LDAK)**		
SNPs restricted for the HapMap3 SNP-set – GCTA heritability model	23.89%	0.021
**Validation model 4: all G43 migraine-diagnosed patients and all controls**	**Heritability on liability scale**	**Corresponding SD ±**
HapMap3 SNPs	12.92%	0.007

Supplementary Table S5 Summarizes the results of heritability estimations performed with SumHer assuming the Human Default Model, on the liability scale and corresponding standard deviations. GWS: genome-wide significant, SD: standard deviation.

Migraine-first patients: initial filtering was based on G43 migraine (with or without aura) diagnosis and narrowed down to migraine-first patients for whom G43 (migraine with or without aura) was their first medical diagnosis in their lifetime.

G43 migraine patients: all migraine-diagnosed patients (G43) without filtering for first onset.

Healthy controls: individuals who had no recorded diseases at the same age when the migraine-first patients received their diagnosis and had no migraine diagnosis in their lifetimeG43 migraine patients: all migraine-diagnosed patients (G43) without filtering for first onset.

All controls: all participants without migraine diagnosis in their lifetime.

2.2. Heritability models

Supplementary Table S6

Heritability Model	Model assumptions
GCTA	$E[h^2_j] = tau_1$; expected heritability is constant across variants
Human Default	$E[h^2_j] = tau_1 [f_j(1-f_j)]^{0.75}$; common variants have higher expected heritability than less common variants
LDAK	$E[h^2_j] = tau_1 w_j [f_j(1-f_j)]^{0.75}$; expected heritability is higher for variants in regions of lower LD and for those with higher MAF
BLD-LDAK	$E[h^2_j] = [f_j(1-f_j)]^{0.75}$ x $(tau_1 b_{1j} + tau_2 b_{2j} + ... + tau_{64} b_{64j} + tau_{65} w_j + tau_{66})$

Supplementary Table S6 shows the underlying assumptions of the different heritability models. $E[h^2_j]$ is the expected heritability, tau_K are the corresponding coefficients, w_j is the weighting for SNP j and f_j is its MAF, b_k are the non-MAF annotation categories.

2.3. Results of different heritability models

Supplementary Table S7

	HD h^2	alt.logl	AIC	GCTA h^2	alt.logl	AIC	LDAK h^2	alt.logl	AIC
WG	0.1937 (0.0197)	-108054.71	216228.84	0.2199 (0.023)	-108279.42	216561.84	0.5282 (0.0481)	-108267.79	216538.57
HP	0.2131 (0.0196)	-69065.03	138133.06	0.2390 (0.0214)	-69018.41	138039.82	0.3314 (0.0297)	-69022.80	138048.52
MAF	0.1903 (0.0200)	-214560.60	429232.24	0.2376 (0.0271)	-214593.20	429189.40	0.5673 (0.0562)	-214582.24	429167.48

Supplementary Table S7 Abbreviations: WG: whole-genome including all variants with MAF ≥ 0.01, HP: only HapMap3 variants, MAF: extended MAF (MAF ≥ 0.001) data, HD: Human Default Model, logl: model fit measured with log-likelihood, AIC: Akaike Information Criterion, h^2 is the estimated heritability and the corresponding standard deviation in brackets.

When considering whole-genome variants, the best-fitting model according to AIC values, is the Human Default, as recommended by the authors of SumHer. However, when restricting the analysis to HapMap3 variants, the GCTA model showed higher model fit, yielding a heritability estimate of 23.90%. When including variants with a broader range of minor allele frequency (MAF ≥ 0.001), the LDAK model provided the best fit, with a heritability estimate of 56.73%, which aligns with twin heritability. The heritability model assumes that heritability is higher for SNPs located in regions of lower linkage disequilibrium (LD) and for those with higher MAF. Consequently, the observed high heritability may be due to inflated estimation, thus, these results should be interpreted with caution. At the same time, model fit suggests that the model assumptions are valid. Further studies need to elucidate if the LDAK model is, indeed, the best fit for estimating migraine heritability in the current setting.

Supplementary Table S8

	BLD-LDAK h^2	alt.logl	AIC
HP	0.2881 (0.0334)	-68984.25	138036.50

Supplementary S8 The estimated heritability for HapMap3 variants was 28.81%. However, these findings should be interpreted with caution due to a warning indicating negative heritability when analyzing a large number of SNPs across different categories. This suggest that in case of out phenotype, this is an overcomplicated heritability model.

Supplementary Figure S1

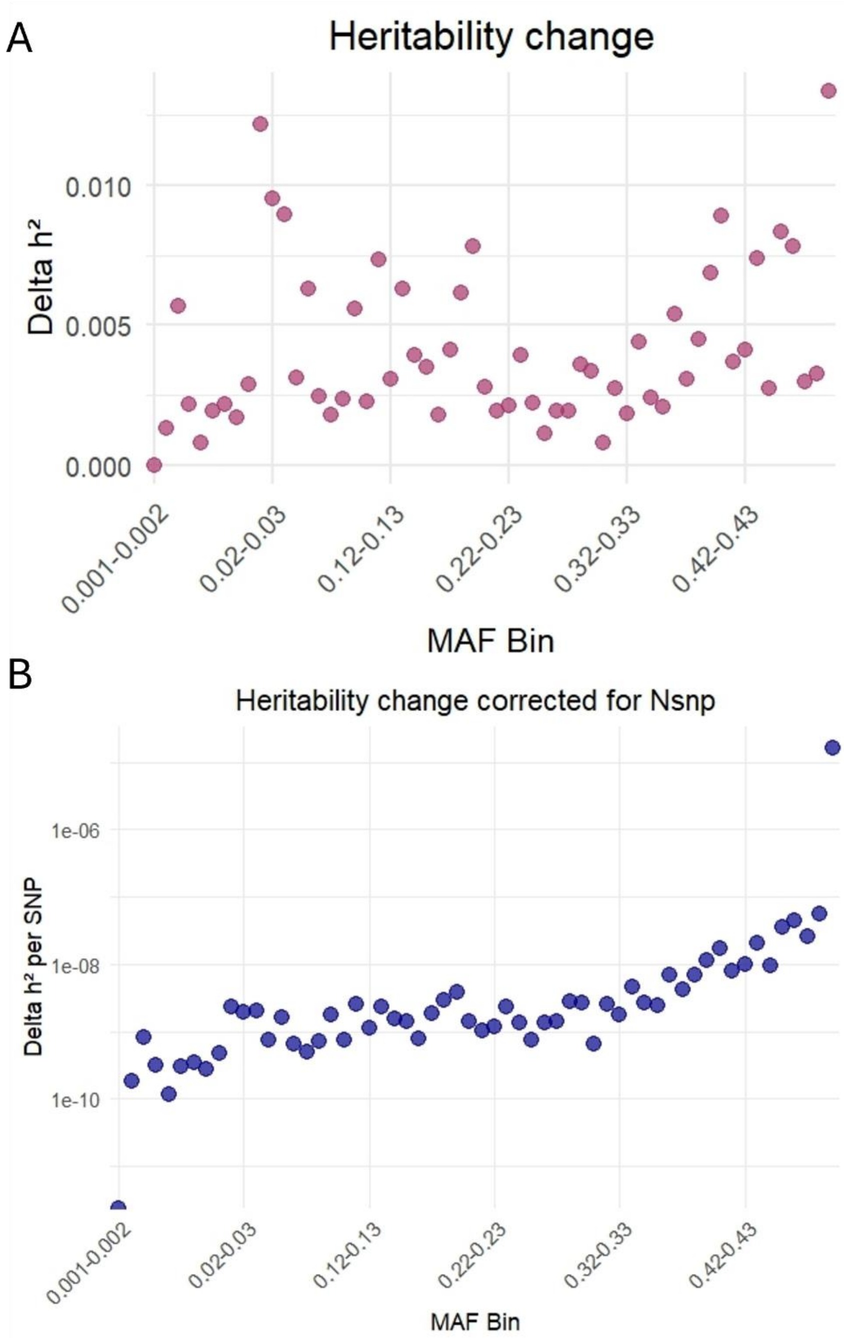

Supplementary Figure S1 shows additional calculations using variants within the 0.01 to 0.001 MAF range. We estimated the heritability contribution of an average variant across 59 different MAF bins, with 0.001 increments between MAF 0.001 and 0.01, and 0.01 increments between MAF 0.01 and 0.5. These computations included both the new variants and those already present in the variant lists. From top-to bottom: **figure A** shows the heritability change between MAF bins. **Figure B** is the heritability change corrected with number of variants in the given bin. (Thus, note, that the lowest MAF bin comprised variants from 0.001-0.002, while the highest was from 0.49-0.50 MAF.)

3. Results of genome-wide association studies of migraine-first patients and healthy controls

3.1. Genome-wide significant SNPs

Supplementary Table S9

Independent significant SNPs	P-value
rs11153082	5.17882e-13
rs7518255	1.23143e-12
rs324012	2.81853e-11
rs11172113	2.83459e-11
rs3798293	4.84526e-11
rs4839683	2.22499e-10
rs12684144	1.46509e-09
rs2651899	3.55413e-09
rs2294897	5.65894e-09

Supplementary Table S9 Genome-wide significant hits ($p < 10^{-8}$) calculated by genome-wide association analysis on migraine-first patients compared to healthy controls.

Migraine-first patients: initial filtering was based on G43 migraine (with or without aura) diagnosis and narrowed down to migraine-first patients for whom G43 (migraine with or without aura) was their first medical diagnosis in their lifetime.

Healthy controls: individuals who had no recorded diseases at the same age when the migraine-first patients received their diagnosis and had no migraine diagnosis in their lifetime.

3.2. Suggestively significant SNPs

Supplementary Table S10

Independent significant SNPs	P-value
rs2455136	3.48298e-08
rs9349379	5.34496e-08
rs6478241	9.37175e-08
rs7396909	1.68409e-07
rs12988953	2.14113e-07
rs2815293	2.37016e-07
rs6709005	4.02961e-07
rs12532479	4.31368e-07
rs10983184	4.47273e-07
rs9381500	4.55036e-07
rs10817892	4.95438e-07
rs113137506	5.41334e-07
rs7045525	7.65819e-07
rs73009672	8.23149e-07
rs10817897	8.27281e-07
rs9359932	9.49267e-07
rs189372686	1.1306e-06
rs6760630	1.18468e-06
rs12202891	1.51512e-06
rs703817	1.56132e-06
rs7394786	1.57408e-06
rs35744865	1.89202e-06
rs6941258	2.00736e-06
rs6854	2.5943e-06
rs11160100	2.63755e-06
rs62527241	2.76753e-06
rs944770	3.2251e-06
rs140119541	3.52334e-06
rs12921494	3.6576e-06
rs6693567	3.71136e-06
rs1342442	3.80485e-06
rs11084993	3.90536e-06
rs7968719	4.40253e-06
rs79101720	4.6571e-06
rs115740972	4.73616e-06
rs79120566	4.81021e-06
rs117637348	5.09348e-06
rs7119658	5.16131e-06
rs11624776	5.25149e-06
rs6046169	5.45211e-06
rs151027228	5.50957e-06
rs2302153	5.64294e-06

rs115756886	5.84691e-06
rs4759272	6.85291e-06
rs117722586	7.15323e-06
rs111936115	7.28096e-06
rs2432301	8.18454e-06
rs112524929	8.60737e-06
rs11220077	8.99632e-06
rs3787078	9.02216e-06
rs10266137	9.14536e-06
rs7148352	9.18899e-06
rs141292640	9.1947e-06
rs116871128	9.4075e-06
rs13429325	9.87643e-06

Supplementary Table S10 Genome-wide and suggestively significant hits ($p < 10^{-5}$) calculated by genome-wide association analysis on migraine-first patients compared to healthy controls.

Migraine-first patients: initial filtering was based on G43 migraine (with or without aura) diagnosis and narrowed down to migraine-first patients for whom G43 (migraine with or without aura) was their first medical diagnosis in their lifetime.

Healthy controls: individuals who had no recorded diseases at the same age when the migraine-first patients received their diagnosis and had no migraine diagnosis in their lifetime.

3.3. Genome-wide significant risk loci

Supplementary Table S11

Chr	Start	End	Independent significant SNPs	P-value	Gene
6	96841762	97067047	rs11153082;rs4839683; rs3798293	5.17882e-13	*FHL5*
1	3065568	3233631	rs7518255;rs2455136; rs2651899	1.23143e-12	*PRDM16*
12	57502182	57534912	rs324012; rs11172113	2.81853e-11	*STAT6, LRP1*
9	119241165	119258583	rs12684144	1.46509e-09	*ASTN2*
20	19455985	19518571	rs2294897	5.65894e-09	*SLC24A3*

Supplementary Table S11 Genome-wide significant risk loci ($p < 10^{-8}$) identified with FUMA by genome-wide association analysis on migraine-first patients compared to healthy controls. These loci are the five loci that were overlapped in all 3 studies (our study, Gormley, and Hautakangas). Chr: chromosome, start: start position of the risk loci, end: end position of the risk loci, Independent significant SNPs: significant independent SNPs located in the risk loci, gene: the annotated genes.

Migraine-first patients: initial filtering was based on G43 migraine (with or without aura) diagnosis and narrowed down to migraine-first patients for whom G43 (migraine with or without aura) was their first medical diagnosis in their lifetime.

Healthy controls: individuals who had no recorded diseases at the same age when the migraine-first patients received their diagnosis and had no migraine diagnosis in their lifetime.

3.4. Genome-wide and suggestively significant risk loci

Supplementary Table S12

Chr	Start	End	Independent significant SNPs	P-value	Gene
6	12768218	12948388	rs9349379;rs9381500;rs12202891	5.34496e-08	***PHACTR1***
11	14980848	15121130	rs7396909;rs7394786	1.68409e-07	*CALCB*
2	234804509	234867513	rs12988953;rs6709005;rs2302153;rs6760630	2.14113e-07	***TRPM8***
7	40360982	40477363	rs12532479	4.31368e-07	***SUGCT/C7orf10***
10	53156442	53222958	rs113137506	5.41334e-07	*PRKG1*
9	86342917	86683120	rs7045525;rs117722586	7.65819e-07	*LOC101927575*
4	166062170	166128741	rs73009672;rs112524929	8.23149e-07	-
6	91892908	91986228	rs9359932	9.49267e-07	-
2	205842583	206337543	rs189372686	1.1306e-06	*PARD3B*
2	231896204	231914845	rs6854	2.5943e-06	*C2orf72*
14	93591673	93596315	rs11160100;rs7148352;rs11624776	2.63755e-06	***ITPK1***
8	106594719	106643913	rs62527241	2.76753e-06	*ZFPM2*
1	206685627	206685627	rs944770	3.2251e-06	*RASSF5*
6	43455719	43633417	rs140119541	3.52334e-06	*POLH, POLR1C*
16	68555187	68680651	rs12921494	3.6576e-06	*ZFP90*
1	150204405	150513711	rs6693567;rs141292640	3.71136e-06	*LOC124904415, RPRD2,* ***ADAMTSL4***
1	156406381	156474929	rs1342442	3.80485e-06	***MEF2D***
19	3042734	3042734	rs11084993	3.90536e-06	*TLE2*
16	79207139	79207139	rs79101720	4.6571e-06	*WWOX*
2	55564300	56101058	rs115740972	4.73616e-06	*PPP4R3B*
2	145755449	145969331	rs79120566	4.81021e-06	*LOC100505498*
7	153143250	153158288	rs117637348	5.09348e-06	-
11	111109258	111111729	rs7119658	5.16131e-06	-
2	224890196	224890196	rs151027228	5.50957e-06	*SERPINE2*
1	100998783	101149093	rs115756886	5.84691e-06	*LOC124904231*
10	26677166	26688963	rs111936115	7.28096e-06	-
16	1056547	1076660	rs2432301	8.18454e-06	-
11	125283916	125456805	rs11220077	8.99632e-06	*FEZ1*
20	25205031	25758201	rs3787078	9.02216e-06	*PYGB*
7	20656054	20724826	rs10266137	9.14536e-06	*ABCB5*
2	66228401	66228539	rs13429325	9.87643e-06	*LINC02934*

Supplementary Table S12 Genome-wide and suggestively significant risk loci ($p < 10^{-5}$) identified with FUMA by genome-wide association analysis on migraine-first patients compared to healthy controls. **Bolded gene names** denote 11 genes which are commonly found in all three GWAS (our study, Gormley and Hautakangas). Chr: chromosome, start: start position of the risk loci, end: end position of the risk loci, Independent significant SNPs: significant independent SNPs located in the risk loci, gene: the annotated genes.

Migraine-first patients: initial filtering was based on G43 migraine (with or without aura) diagnosis and narrowed down to migraine-first patients for whom G43 (migraine with or without aura) was their first medical diagnosis in their lifetime.

Healthy controls: individuals who had no recorded diseases at the same age when the migraine-first patients received their diagnosis and had no migraine diagnosis in their lifetime.

3.5. GTEx v8 significant tissue type

Supplementary Table S13

Tissue type	P-value
Artery Tibial	0.0001927

Supplementary Table S13 Significant tissue-specific enrichment of our risk loci using suggestive significance SNP threshold ($p < 10^{-5}$) calculated with FUMA by genome-wide association analysis on migraine-first patients compared to healthy controls.

Migraine-first patients: initial filtering was based on G43 migraine (with or without aura) diagnosis and narrowed down to migraine-first patients for whom G43 (migraine with or without aura) was their first medical diagnosis in their lifetime.

Healthy controls: individuals who had no recorded diseases at the same age when the migraine-first patients received their diagnosis and had no migraine diagnosis in their lifetime.

3.6. Gene-set enrichment analysis

The performed MAGMA analyses show whether the genes of interest (based on GWAS) are overrepresented in any of the pre-defined gene sets from MSigDB C2 – KEGG pathways and C3 - Transcription Factor Targets (TFT). MSigDB: Molecular Signatures Database: (https://www.gsea-msigdb.org/gsea/msigdb/collections.jsp).

3.6.1. MsigDB C2 – KEGG

Supplementary Figure S2

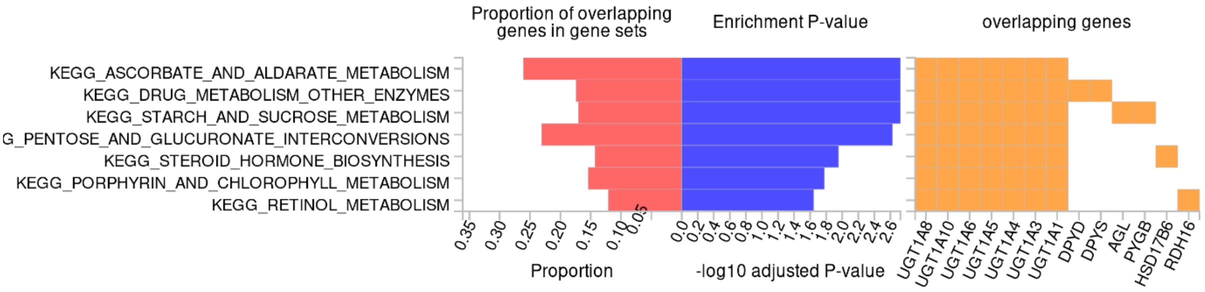

Supplementary Figure S2 Seven KEGG pathways (MsigDB C2 sets) as the result of MAGMA gene-set enrichment analysis, by genome-wide association analysis on migraine-first patients compared to healthy controls. KEGG: Kyoto Encyclopedia of Genes and Genomes (https://www.genome.jp/kegg/pathway.html).

Migraine-first patients: initial filtering was based on G43 migraine (with or without aura) diagnosis and narrowed down to migraine-first patients for whom G43 (migraine with or without aura) was their first medical diagnosis in their lifetime.

Healthy controls: individuals who had no recorded diseases at the same age when the migraine-first patients received their diagnosis and had no migraine diagnosis in their lifetime.

3.6.2. MsigDB C3 – Transcription Factor Targets

Supplementary Figure S3

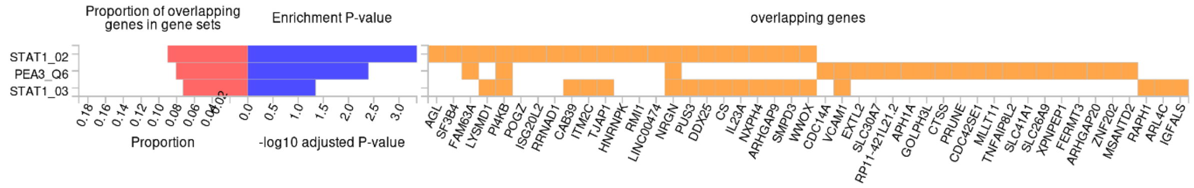

Supplementary Figure S3 Shows three significant transcription factor-related sets (MsigDB-C3) as the result of MAGMA gene-set enrichment analysis, by genome-wide association analysis on migraine-first patients compared to healthy controls.

Migraine-first patients: initial filtering was based on G43 migraine (with or without aura) diagnosis and narrowed down to migraine-first patients for whom G43 (migraine with or without aura) was their first medical diagnosis in their lifetime.

Healthy controls: individuals who had no recorded diseases at the same age when the migraine-first patients received their diagnosis and had no migraine diagnosis in their lifetime.
